# Type I beta turns make a new twist in pentapeptide repeat proteins: Crystal structure of Alr5209 from Nostoc sp. PCC 7120 determined at 1.7 angström resolution

**DOI:** 10.1016/j.yjsbx.2019.100010

**Published:** 2019-08-14

**Authors:** Ruojing Zhang, Shuisong Ni, Michael A. Kennedy

**Affiliations:** Department of Chemistry and Biochemistry, Miami University, Oxford, OH 45056, United States

**Keywords:** Pentapeptide repeat protein, Repeat five residue fold, Beta turns, Cyanobacteria, Protein crystal structure, Nostoc sp. PCC 7120

## Abstract

Pentapeptide repeat proteins (PRPs) are found abundantly in cyanobacteria, numbering in the dozens in some genomes, e.g. in Nostoc sp. PCC 7120. PRPs, comprised of a repeating consensus sequence of five amino acids, adopt a distinctive right-handed quadrilateral β-helical structure, also referred to as a repeat five residue (Rfr) fold, made up of stacks of coils formed by four consecutive pentapeptide repeats. The right-handed quadrilateral β-helical PRP structure is constructed by repeating β turns at each of four corners in a given coil, each causing a 90° change in direction of the polypeptide chain. Until now, all PRP structures have consisted either of type II and IV β turns or exclusively of type II β turns. Here, we report the first structure of a PRP comprised of type I and II β turns, Alr5209 from Nostoc sp. PCC 7120. The *alr5209* gene encodes 129 amino acids containing 16 tandem pentapeptide repeats. The Alr5209 structure was analyzed in comparison to all other PRPs to determine how type I β turns can be accommodated in Rfr folds and the consequences of type I β turns on the right-handed quadrilateral β-helical structure. Given that Alr5209 represents the first PRP structure containing type I β turns, the PRP consensus sequence was reevaluated and updated. Despite a growing number of PRP structural investigations, their function remains largely unknown. Genome analysis indicated that *alr5209* resides in a five-gene operon (*alr5208*-*alr5212*) with Alr5211 annotated to be a NADH dehydrogenase indicating Alr5209 may be involved in oxidative phosphorylation.

## Highlights

• Alr5209 is the first pentapeptide repeat protein that includes type I beta turns.
• PRPs containing type I beta turns have the tightest beta helical coil turn radius.
• PRPs containing type IV beta turns have the largest right-handed beta helix diameters.
• PRPs containing mixed type IV and type I or II beta turns have negative coil twist.

## Graphical abstract

**Figure:**
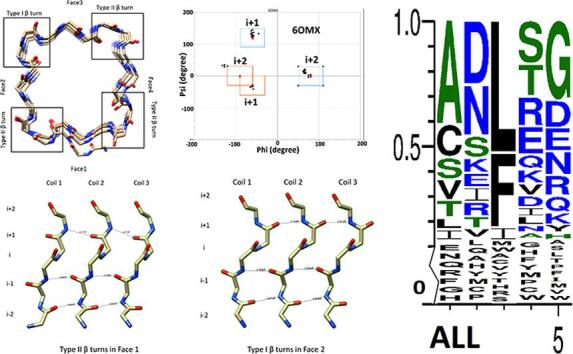

## 1 Introduction

Cyanobacteria, ancient prokaryotic microorganisms capable of both oxygenic photosynthesis and nitrogen fixation, are thought to be the first organisms responsible for oxygenation of the earth’s atmosphere more than two billion years ago (Ecology of Cyanobacteria II, 2012, Giovannoni et al., 1988, Black et al., 1995). In the filamentous *Nostoc* sp. Strain PCC 7120 cyanobacterium, the filaments can grow to contain several hundred cells due to division of actively dividing vegetative cells (Herrero et al., 2016). Nitrogen fixation in *Nostoc* sp. PCC 7120 takes place in specialized cells known as heterocysts (Herrero et al., 2016) that differentiate from vegetative cells under conditions of low available nitrogen. Under such conditions, 5 to 10% of the vegetative cells in the filament in *Nostoc* sp. PCC 7120 differentiate into heterocysts, with adjacent heterocysts regularly spaced by about ten vegetative cells (Golden and Yoon, 2003), thus providing a source of nitrogen to the surrounding vegetative cells in the filament. Both the vegetative cells and heterocysts in filaments of *Nostoc* sp. PCC 7120 are capable of performing multiple functions to adapt to changing conditions in their surroundings. The adaptability of *Nostoc* sp. PCC 7120 to its environment requires both vegetative and heterocyst cells to carry out many biochemical functions including photosynthesis, nitrogen fixation, signal communication and cell differentiation (Wang et al., 2002). In 2001, the complete genome of *Nostoc* sp. PCC 7120, containing a 6.4 Mb chromosome and six plasmids, was sequenced and 6228 proteins were predicted to be encoded by the chromosome. Given the availability of its complete genome sequence and the fact that filamentous cyanobacteria represent among the oldest and simplest living organisms to exhibit cell differentiation (Hamilton et al., 2016); *Nostoc* sp. PCC 7120 has become an important model organism to study biochemical functions found in cyanobacteria (Kaneko et al., 2001).

Pentapeptide repeat proteins (PRPs) represent a large superfamily of proteins with 52,787 sequences grouped into four clans in the Pfam database (El-Gebali et al., 2019). Analysis of the largest PRP clan, represented by the Pentapeptide family (Pfam 00805), that includes 38,471 sequences from 3485 species indicates that ~90% of the sequences belong to bacteria and archaea while ~10% of sequences belong to eukaryotes. Further analysis indicates that nearly half of the PRP sequences in bacteria belong to cyanobacteria and that PRPs are most abundant in cyanobacteria in terms of the numbers of PRPs per genome (Ni et al., 2009). PRPs, defined as proteins containing at least eight tandem repeating sequences of five amino acids with a consensus sequence originally defined as A[D/N]LXX in 1998 (Bateman et al., 1998), also referred to here as PRP domains, adopt a distinctive right-handed β-helical solenoid structure composed of stacks of coils composed of four pentapeptide repeats. Thirty PRPs have been identified in *Nostoc* sp. PCC 7120, including HglK (All0813), a membrane protein reported to be involved to the localization of heterocyst-specific glycolipids (Black et al., 1995). In 2009, the structure of HetL, a PRP from *Nostoc* sp. PCC 7120 containing 40 tandem repeats involved in regulating differentiation of heterocysts, was reported (Ni et al., 2009). Despite the important role that cyanobacteria played in evolution of the earth's atmosphere and oxygen-based life on earth, and the relative abundance of PRPs in cyanobacteria, the biochemical functions of PRPs remain largely unknown and only sixteen PRP structures have been reported (Ni et al., 2011, Diao et al., 2008, Lin et al., 2011, Yao et al., 2016, Benoit et al., 2014).

In this study, we determined the structure of Alr5209, a PRP found in *Nostoc* sp. PCC 7120. The structure adopts a repeat five residue (Rfr) fold composed of 16 tandem PRP domains. The resulting right-handed β helix is composed of four coils held together by β ladders composed of β bridges on each face and a 1:3 mixture of type I and type II β turns. Alr5209 is the first PRP reported to contain type I β-turns in its Rfr fold. The structural consequences of including type I turns in the Rfr fold are examined and discussed. Combined structure and sequence analysis of Alr5209 enabled refinement of the pentapeptide consensus sequences that encode PRPs, which should allow for more sensitive and accurate prediction of PRPs in existing and newly reported genomes. Finally, a gene cluster analysis based on the KEGG database indicated that Alr5209 may be involved in oxidative phosphorylation.

## 2 Materials and methods

### 2.1 Cloning, expression and purification

The *alr5209* gene was amplified from the genomic DNA of *Nostoc* sp. PCC 7120 using standard PCR methods. Based on analysis of the KEGG sequence for *alr5209*, the following two primers were designed containing Ndel and Xhol ligation sites to facilitate construction of the expression plasmid: cccgcccgcatATGTCTGAAGTCAATTATCAACAG and gcccgctcgagttaTTGTTCTTTGAGTTGCAAGCC. The PCR product was cloned into the pET28b expression vector (Novagen, Inc.) under the control of the T7 promoter, and the construct contained a N-terminal 6xHis tag to allow purification by nickel affinity chromatography. The constructed plasmid was transformed into JM109 competent cells (Novagen, Inc.), spread on agar plates and resulting colonies collected for sequencing. After sequencing confirmed successful cloning of the *alr5209* gene into the expression plasmid, the plasmid was transformed into the *Escherichia coli* BL21 (DE3) (Novagen, Inc) host strain for overexpression of Alr5209 protein. Protein was isolated from a one-liter culture grown in M9 minimal medium using N15-labeled ammonium chloride as a nitrogen source to enable isotopic labeling for future nuclear magnetic resonance spectroscopy experiments. Cell growth in the bacterial culture was maintained at 37 °C with 250 revolutions per minute (rpm) shaking until the OD_600_ reached to 0.6–0.8. At this point, the cell culture was cooled to 15 °C and 0.5 mL 1 M isopropyl β-D-1-thiogalactopyranoside (IPTG) was added to a final concentration of 0.5 mM. The culture was then incubated at 15 °C with 250 rpm shaking overnight. The cells were collected using 5000×*g* centrifugation at 4 °C for 20 min. The resulting cell pellet was resuspended in 20 mL B1 buffer (20 mM Tris, 250 mM NaCl, 10% glycerol, pH 7.8) and the resuspended cells were lysed by three passes through a French press (Thermo, Inc.). The cell lysate was centrifuged at 17,418×*g* for 30 min. The His-tagged protein in the supernatant was purified on a 20 mL Ni-NTA affinity column (Qiagen). Proteins in the supernatant lacking a His-tag were removed during successive washing steps with 60 mL B1 buffer containing 0 and 30 mM imidazole, respectively. The purified His-tagged Alr5209 protein eluted with 300 mM imidazole was then dialyzed three times with 1 L B1 buffer to remove imidazole. Purified Alr5209 protein was confirmed by SDS-PAGE gel and concentrated to final concentration of 35 mg/mL.

### 2.2 Crystallization, data collection, phasing and refinement

Crystallization conditions were determined using the Hampton Research kit (HR2-112 and HR2-121) to screen for protein crystallization. Screening was performed by combining 1 μL of protein with 1 μL of each buffer on a 48-well plate using the hanging-drop vapor-diffusion method. Plates were maintained at room temperature. Overlapped spherical crystals were obtained in a buffer containing 0.2 M potassium sodium tartrate tetrahydrate, 0.1 M sodium citrate tribasic dihydrate pH 5.6, 2.0 M ammonium sulfate. These crystals were crushed in 50 μL crystallization buffer using a crystal crusher and by glass beads to make a stock seeding solution. Final cubic crystals were obtained by adding 0.5 μL of a 10,000× diluted seeding solution to 1 μL protein and 1 μL cryo-buffer, consisting 0.15 M potassium sodium tartrate tetrahydrate, 0.075 M sodium citrate tribasic dihydrate pH 5.6, 1.5 M ammonium sulfate, 25% v/v glycerol.

All experiment diffraction data were collected at the Advanced Photon Source (APS) at Argonne National Laboratory using the beamline 31-ID at 100 K. Truncated I to F experimental data analyzed by CCP4 7.0.057 were submitted to the CCP4 online server and a molecular replacement solution was found by BALBES (Long et al., 2008) using the PDB ID 2J8I structure as a starting model (Vetting et al., 2007). Manual model building was performed using COOT (Emsley et al., 2010). Phenix 1.13 (Adams et al., 2010) was used for phasing improvement, automatic amino acid building and refinement. The final structure was submitted to the Protein Data Bank (PDB ID: 6OMX). The electrostatic potential surface was calculated using the PDB2PQR server (Dolinsky et al., 2004) and depicted using the Chimera software (Pettersen et al., 2004).

### 2.3 Secondary structure and sequence analysis

Distances in the PRPs and the φ and ψ angles were measured using Chimera (Pettersen et al., 2004). All distance measurements were performed on PRPs with known structures and structure-based sequence alignment starting from the first, N-terminal pentapeptide repeat domain. The face of the right-handed β-helices containing the first, N-terminal complete pentapeptide repeat domain was designated as face 1, except for 3PSS whose first pentapeptide in coil 1 was incomplete. The face of the right-handed β-helices containing the second pentapeptide repeat domain was designated as face 2, and so on. The β turn types and distributions were measured from the PDB coordinates of published structures. The length of each face was measured from the carbonyl carbon of the i − 2 amino acid to that of the i + 2 amino acid for each face. The face-to-face distances between the 1 and 3 faces were measured from the carbonyl carbon of the i-residue in face 1 to the carbonyl carbon of the i residue in face 3. The face-to-face distances between the 2 and 4 faces were measured from the carbonyl carbon of the i-residue in face 2 to the carbonyl carbon of the i-residue in face 4. The distances across the face 1 to face 2 turns were measured from the carbonyl carbon of the i-residue in face 1 to the carbonyl carbon of the i-residue in face 2. The distances across the face 1 to face 4 turns were measured from the carbonyl carbon of the i-residue in face 1 to the carbonyl carbon of the i-residue in face 4. Any PRP coils interrupted by an inner loop or other secondary structures rather than β helix were not counted in the summary statistics. Consensus sequence distribution plots were completed using the Web Logo server (Crooks et al., 2004, Schneider and Stephens, 1990). Sequences belonging to secondary structures other than the PRP domains were not included in the consensus sequence analysis. For calculation of twist angle among coils, the angle calculation tool in Chimera was used (Pettersen et al., 2004). Twist angles were measured as the angle between the two vectors defined by the carbonyl carbons of the i − 2 and i + 2 amino acids from coil and the carbonyl carbons of the i − 2 and i + 2 amino acids of the following coil. Once those vectors were defined based on those two pairs of atoms, the twist angles were determined using the angle calculation tool. Due to the influence of an α helix near the N-terminus, the twist angles in 6OMX, 2J8K, 3PSS and 3DU1 were measured between the second coil and subsequent coils.

### 2.4 Circular dichroism (CD) spectroscopy and thermal protein denaturation

Purified protein was dialyzed and diluted to final concentration at 20 μM with 20 mM potassium phosphate pH 7.8 and 150 mM NaF buffer. Diluted protein samples were loaded into 1 mL quartz cuvettes. Experiments were performed with AVIV model435 circular dichroism spectrophotometer (Aviv Biomedical, Inc). Far-UV wavelength spectra were recorded from 180 nm to 300 nm to determine a suitable wavelength for temperature melting experiments at 25 °C. Thermal denaturation curves for 20 μM samples were collected both at 226 nm and 210 nm, separately, from 15 to 85 °C using 1 °C intervals. Wavelength scans were measured for both samples at 85 and 95 °C after the thermal denaturation experiments. Experiments with buffer only were performed under the same conditions as with the protein samples and used as blanks for correction. Data analysis of thermal denaturation experiments was performed using the Calfitter 3.1 software package using the natured-state equilibrium with denatured-state (N = D) model for fitting (Mazurenko et al., 2018).

## 3 Results and discussion

### 3.1 Crystal and data quality of Alr5209

Original crystals were spherical and overlapping. High-quality single crystals suitable for X-ray diffraction measurements were obtained using seeding and addition of glycerol. Crystals used for diffraction data collection were orthorhombic (unit cell dimensions: a = 71.001 Å, b = 27.835 Å, c = 60.837 Å, α = β = γ = 90°) and the space group was P222_1_. Single wavelength data collected at 0.97931 Å was used for molecular replacement. The data was truncated to 1.71 Å with an overall completeness of 98.71% measured for 13,598 unique reflections. Xtriage (Zwart et al., 2005a, Zwart et al., 2005b) analysis indicated a single molecule in the asymmetric unit with a solvent content of 0.407. Molecular replacement phasing was accomplished using 2J8I as a starting model. The final structure included 121 out of 129 amino acids with six residues missing at the N-terminus and two residues missing at the C-terminal end. The structure quality was checked using MolProbity (Chen et al., 2010) and the PDB validation server. The report showed no Ramachandran outliers and clash scores and sidechain outliers were 2 and 2.1%, respectively. All data and refinement statistics are listed in Table 1.

**Table 1:** Summary of data collection and structure refinement data for alr5209.

Resolution range (Å)	35.5–1.706 (1.767–1.706)^1^
Space group	P222_1_
Unit cell (Å, °)	α = 71.001 β = 27.835 γ = 60.837 α = β = γ = 90
Total reflections	27,104 (2671)
Unique reflections	13,598 (1339)
Multiplicity	2.0 (2.0)
Completeness (%)	98.70 (99.18)
Mean I/sigma (I)	21.77 (2.49)
Wilson B-factor (Å^2^)	28.67
R-merge	0.00942 (0.2226)
R-means	0.01332 (0.3148)
R-pim	0.00942 (0.2226)
CC1/2	1 (0.91)
CC*	1 (0.976)
Reflections used in refinement	13,583 (1335)
Reflections used for R-free	1359 (133)
R-work	0.2149 (0.3316)
R-free	0.2489 (0.3837)
CC (work)	0.974 (0.777)
CC (free)	0.957 (0.678)
Number of non-hydrogen atoms	938
macromolecules	925
solvent	13
Protein residues	121
RMS (bonds) (Å)	0.010
RMS (angles) (°)	1.39
Ramachandran favored (%)	98.32
Ramachandran allowed (%)	1.68
Ramachandran outliers (%)	0.00
Rotamer outliers (%)	0.00
Clashscore	1.09
Average B-factor (Å^2^)	47.00
Macromolecules (Å^2^)	46.99
Solvent (Å^2^)	48.18
Number of TLS groups	1

1 Statistics for the highest-resolution shell are shown in parentheses*.*

### 3.2 Structure analysis of Alr5209

**Fig. 1:**
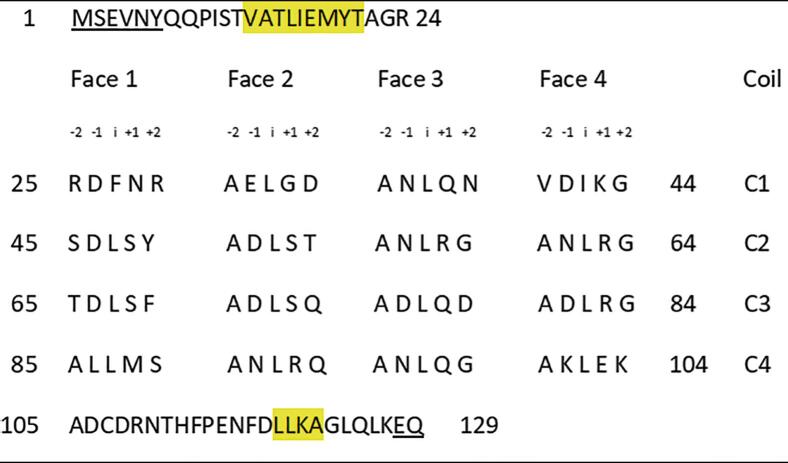
Alignment of the PRP domains in Alr5209 based on its structure. Underlined residues were not visible in the electron density and were not modeled, α-helical residues are highlighted in yellow. Residues 25 to 104 comprised the pentapeptide repeat domains defining the Rfr solenoid.

**Fig. 2:**
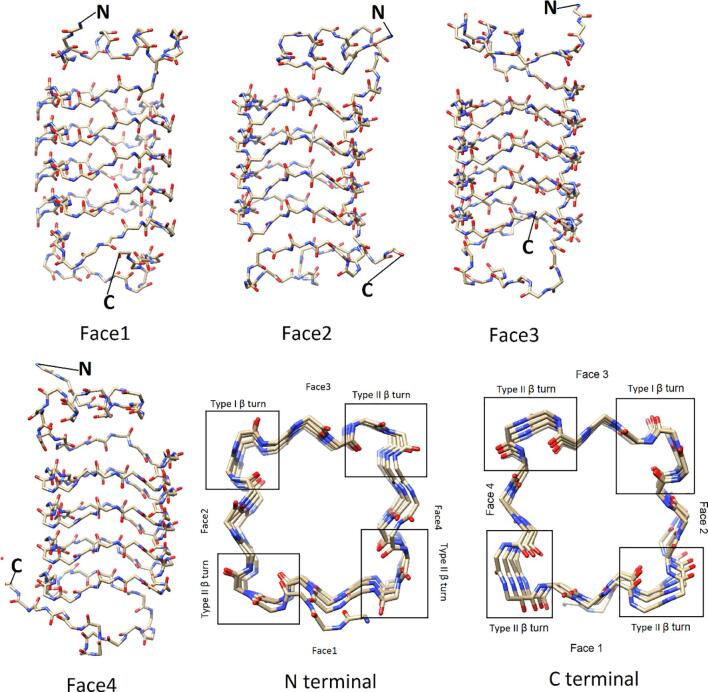
Overview of the backbone structure in the Rfr fold of Alr5209. The four faces of the Alr5209 PRP structure are depicted using a stick representation colored by heteroatom type. N- and C-termini are labeled in each representation. The two on-axis views are depicted at the lower right excluding the α-helix facing the viewer for clarity. The type of β-turn type was labeled for the on-axis views.

**Fig. 3:**
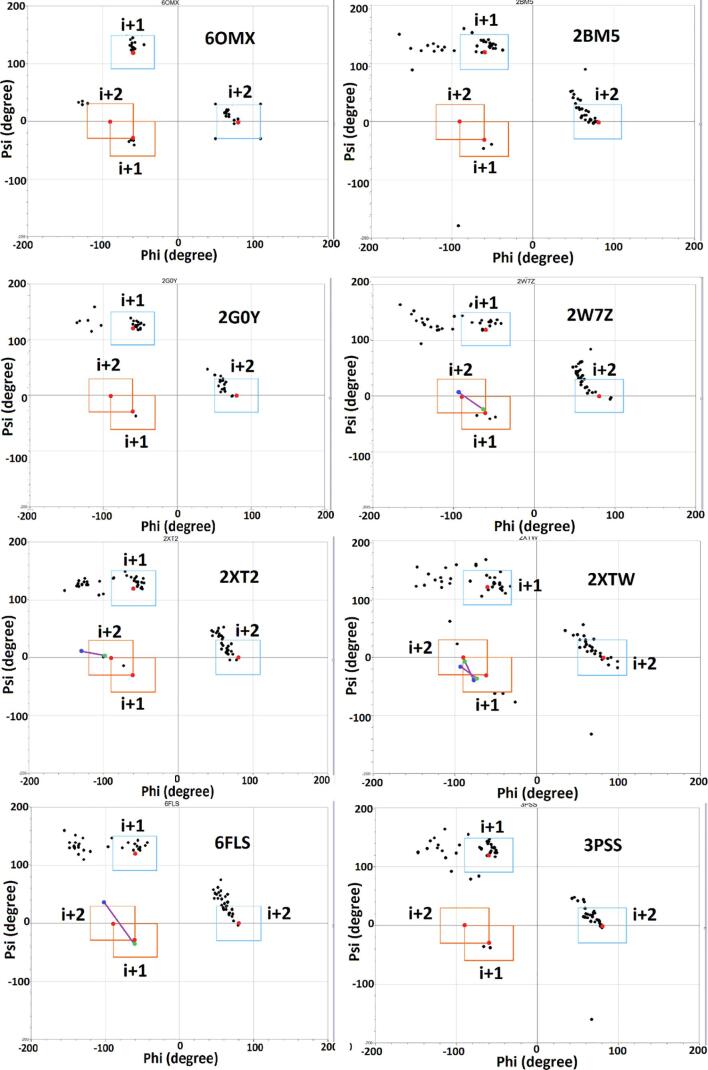
Ramachandran plot of type I and type II β turns in Alr5209 in comparison to other PRPs. The orange boxes indicate canonical values (red points) ±30° for type I β turns. The blue boxes indicate canonical values (red points) ±30° for type II β turns. Except 6OMX, all type I/IV β turns locating in or near orange part are linked by purple lines, the blue points stand i + 2 and the green points are i + 1. (For interpretation of the references to color in this figure legend, the reader is referred to the web version of this article.)

Alr5209 contained 16 pentapeptide repeat domains (Fig. 1) that formed a right-handed quadrilateral β helix consisting of a stack of four Rfr coils with α-helices at the N- and C-termini (Fig. 2). The N-terminal α-helix contained nine amino acids (13-VATLIEMYT-21) while the C-terminal α-helix was shorter being comprised of four amino acids (119-LLKA-122). The Rfr folds of PRPs are constructed by four β-turns per coil with the type of β-turn being defined by combinations of φ and ψ angles of the residues involved in making up the turns (Shapovalov et al., 2019, Hutchinson and Thornton, 1994). Type I and II β turns are distinguished by differences in the ψ angle in the i + 1 position and the φ angle in the i + 2 position with canonical type II β turns having φ and ψ angles of +80° and +120° in these positions, respectively (Hutchinson and Thornton, 1994) (Table 2). Based on the analysis of φ and ψ angles, Alr5209 is composed of a mixture of type I and type II β turns (Table 2). The type I β turns in Alr5209 appeared in every coil in the same position (joining face 2 and face 3) and the rest of the turns were type II β turns. In the i + 1 position of face 2, the φ and ψ angles were −61 ± 3° and −35 ± 4° consistent with the canonical definition of type I β turns (−60°/−30°) (Shapovalov et al., 2019, Hutchinson and Thornton, 1994). In the i + 2 position of face 2, the φ and ψ angles were −127 ± 4° and 32 ± 2°, whereas the the φ and ψ angles of the i + 2 residues in that canonical definition of type I β turns are −90° and 0°, respectively. Therefore, the φ and ψ angles of the i + 2 residues were −/+30° from the canonical type I values, respectively, putting them just outside the edge of canonical values used to define type I β turns (Fig. 3) (Shapovalov et al., 2019). While all other PRPs contain combinations of type II and type IV β turns, Alr5209 is the only known PRP that contains exclusively type I β turns in the same corner of its Rfr solenoid (Fig. 3). Close inspection of the graphs in Fig. 3 reveals that three PRPs classified as containing mixtures of type II and type IV β turns contain one (2W7Z and 6FLS) or two (2XTZ) type I β turns, respectively. The remaining PRPs classified as containing mixtures of type II and type IV β turns (2BM5, 2G0Y, 2XT2 and 3PSS) did not contain any β turns that could be classified as type I β turns.

**Table 2:** Summary of φ and ψ angles for each amino acid position in the PRP domains in Alr5209.

	Face 1		Face 2		Face 3		Face 4	
	φ (°)	ψ (°)	φ (°)	ψ (°)	φ (°)	ψ (°)	φ (°)	ψ (°)
i − 2	−4 ± 75	112 ± 73	−70 ± 9	147 ± 7	−71 ± 3	146 ± 2	−74 ± 3	150 ± 5
i − 1	−93 ± 6	109 ± 5	−100 ± 5	102 ± 5	−91 ± 3	107 ± 3	−103 ± 8	107 ± 3
i	−117 ± 7	23 ± 7	−123 ± 6	34 ± 2	−115 ± 4	25 ± 7	−118 ± 7	23 ± 11
i + 1	−56 ± 7	135 ± 5	−61 ± 3	−35 ± 4^2^	−61 ± 1	128 ± 3	−60 ± 3	135 ± 7
i + 2	64 ± 2	15 ± 4	−127 ± 4^2^	32 ± 2	68 ± 4	10 ± 9	72 ± 6	8 ± 6

2 these φ and ψ angles distinguish between type I and II β turns.

**Fig. 4:**
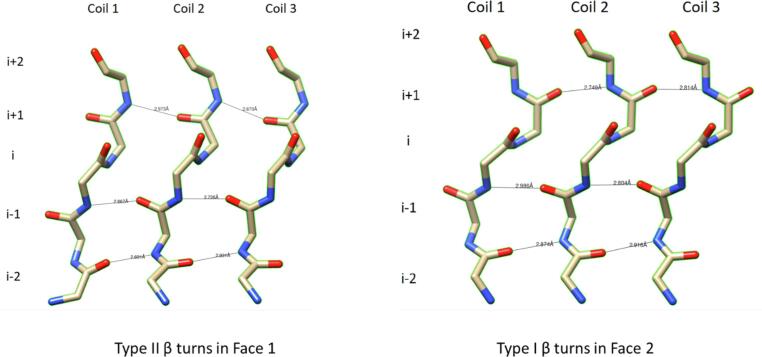
Details of type I and type II β turns in Alr5209. The β turns are defined by residues in the i + 2, i + 1, i and i − 1 positions in PRPs. The difference in the combination of the ϕ and Ψ angles that distinguish the type I and type II turns results in a change in the direction of the hydrogen bonds formed between i + 1 and i + 2 residues involved in stabilizing the intercoil structure involving type II turns (left) and type I turns (right).

Analysis of the Alr5209 structure showed that the direction of the inter-coil hydrogen bond linkages that establish the β-bridges in type I β turns were different in i + 1 and i + 2 positions compared to in the type II β turns. In both type I and type II β turns, the i + 1 carbonyls always acted as hydrogen bond acceptors and the i + 2 amide groups always acted as the hydrogen bond donors. However, in the type II β turns, the i + 1 amino acid carbonyl hydrogen bond acceptor is always on the coil C-terminal to the coil containing the i + 2 amino acid amide hydrogen bond donor (Fig. 4). In contrast, in type I β turns, the linkage of hydrogen bonds establishing the β-bridges is flipped with the i + 2 amino acid amide hydrogen bond donor always in the coil C-terminal to the coil containing the i + 1 amino acid containing the carbonyl hydrogen bond acceptors (Fig. 4).

### 3.3 Electrostatic potential surface of Alr5209

**Fig. 5:**
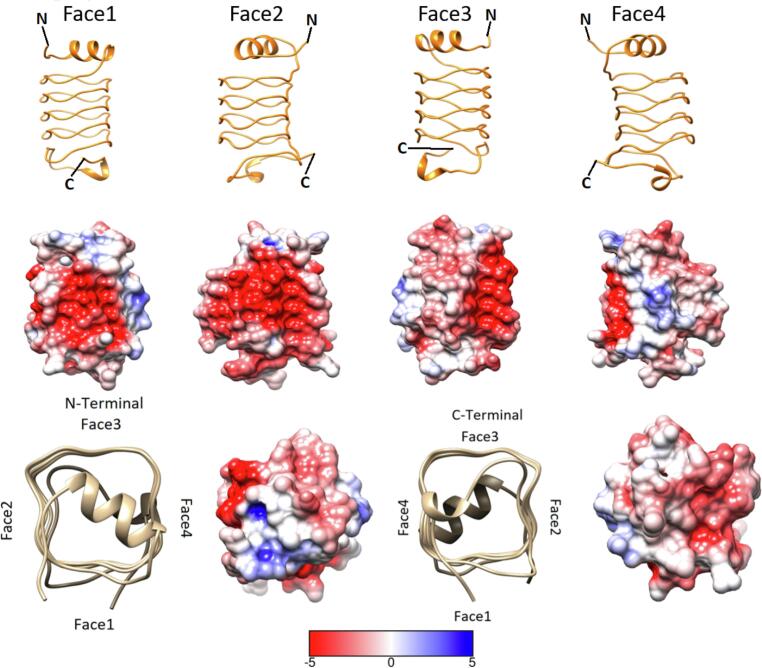
Electrostatic surface potential of Alr5209 for each face of the right-handed quadrilateral β helix. The electrostatic surface potential surface is depicted for each of the four faces. The Rfr fold coil structure is depicted above each electrostatic potential plot for reference. Red indicates negative charge and blue indicates positive charge with the relative intensity indicated by the scale bar at the bottom. The electrostatic potential at the N-terminus and C-terminus of the right-handed β helix is depicted at the bottom using two on-axis plots. The scale for the surface potential color gradient has units of kT/e where 1 kT/e = 25.7 mV. (For interpretation of the references to color in this figure legend, the reader is referred to the web version of this article.)

The electrostatic potential surface of Alr5209 is shown in Fig. 5. Faces 1 and 2 were dominated by strong negative charge whereas face 3 showed a mostly neutral charge distribution and face 4 showed predominantly positive charge (Fig. 5). The C-terminal surface was neutral and the N-terminal surface contained a mixture of positive, negative and neutral charge distribution. This charge distribution would be consistent with functioning as a DNA mimic which has been reported for the fluoroquinolone resistance protein from *Mycobacterium tuberculosis* (Hegde et al., 2005).

### 3.4 Circular dichroism spectroscopy analysis of the Alr5209 structure and thermal stability

**Fig. 6:**
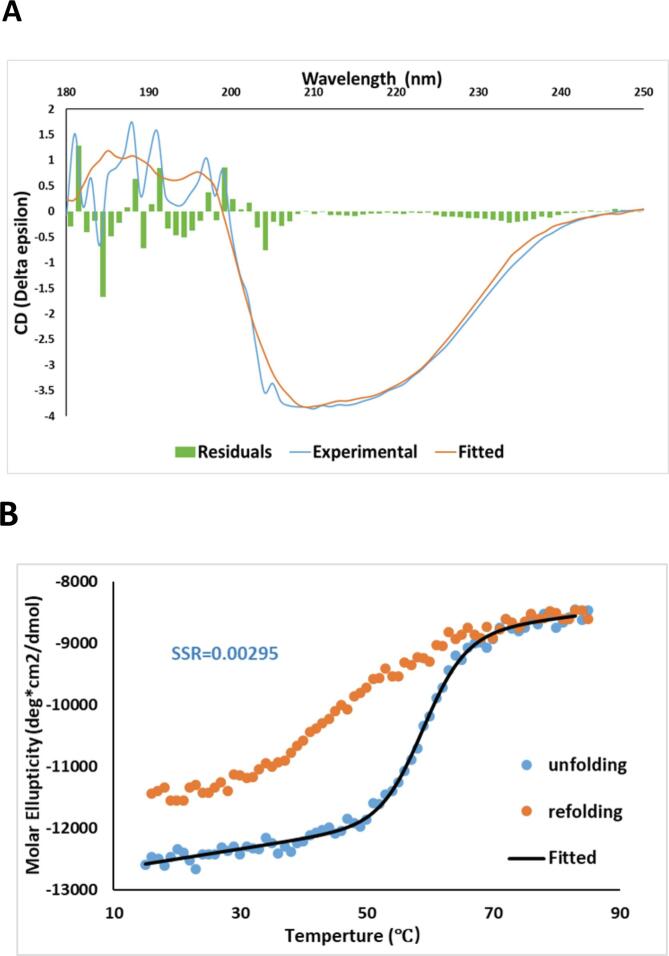
CD spectrum and temperature melting experiments for Alr5209. A) Wavelength scan for 20 μM protein at 25 °C depicted with buffer scan correction and fitted curve. The peak in ellipticity occurred at 210 nm. B) Graph of data points for the temperature melting experiments measured from 15 °C to 85 °C recorded at 210 nm. Increasing temperature from 15 °C to 85 °C resulted in unfolding of the protein and subsequent decreasing of the temperature from 85 °C to 15 °C allowed protein refolding. The degrees of freedom used for fitting was 64. SSR = sum of squared residuals.

The thermodynamic stability of the right-handed quadrilateral β-helical structure of Alr5209 was investigated by CD-monitored thermal melting analysis. The room-temperature CD spectrum was consistent with a structure dominated by type 1 and type II β-turns with short N-terminal and C-terminal α-helices (Fig. 6). At 25 °C, the strongest ellipticity appeared at 210 nm, which could be fit with a composition of secondary structural components consisting of 21.9% α-helix, 13% turn, antiparallel and parallel β-sheet occupy 13.1% and 4.5%, respectively (Fig. 6A, Table 3). Lack of perfect fitting may reflect an incomplete basis set, for example, lack of characteristic CD contributions of type I and type II β turns in PRP structures. The CD melting experiment (Fig. 6B) indicated melting temperature of Alr5209 was 59.0 ± 0.7 °C. Compared to the average melting temperature of 62.1 ± 15.0 °C reported for a distribution of over 1100 proteins (Bava et al., 2004), the melting temperature of Alr5209 fell within the average range (Xu et al., 2017). The reverse melting experiment indicated that denatured alr5209 could be mostly refolded (73.8%) after thermal denaturation (Greenfield, 2006). Alr5209’s melting temperature was ~3 °C lower compared to that of At2g44920 (Xu et al., 2017). The longer hydrogen bonding network in the extended Rfr coil structure of At2g44920 could require substantially more thermal energy to denature the overall right-handed quadrilateral β-helical structure compared to the Alr5209 structure, which contains only two internal Rfr coils sandwiched by two terminal Rfr coils. The enthalpy of unfolding of Alr5209 was −64.6 ± 8.8 kcal/mol, was smaller and of opposite sign compared to that of At2g44920, which was reported to be +120 kcal/mol.

**Table 3:** The table summarizes the secondary structure contributions to the fit.

Helix	21.9%	Helix1 (regular)	6.5%
		Helix2 (distorted)	15.5%
Antiparallel	13.1%	Anti1 (left-twisted)	0%
		Anti2 (relaxed)	1.7%
		Anti3 (right-twisted)	11.4%
Parallel	4.5%		
Turn	13%		
Others	47.5%		

### 3.5 Insight into potential function of Alr5209 from gene cluster analysis

A gene cluster analysis based on the Kyoto Encyclopedia of Genes and Genomes (KEGG) database (Howitt et al., 1999) indicated that five genes, including *alr5209*, belong to the same operon. Of those genes (*alr5208, Alr5209, alr5210, alr5211* and *alr5212*), *alr5208, alr5209*, and *alr5212* were annotated as hypothetical proteins with unknown functions. Alr5210 was annotated as a two-component hybrid sensor and regulator but its function is still unknown and Alr5211 was recognized as a NADH dehydrogenase involved in oxidative phosphorylation based on analogy to the gene cluster with *slr0851, slr1743*, and *sll1484* in cyanobacterium *Synechocystis* sp strain PCC 6803, therefore, Alr5209 may be involved in oxidative phosphorylation (Howitt et al., 1999).

### 3.6 Re-examination of PRP domain consensus sequences

**Fig. 7:**
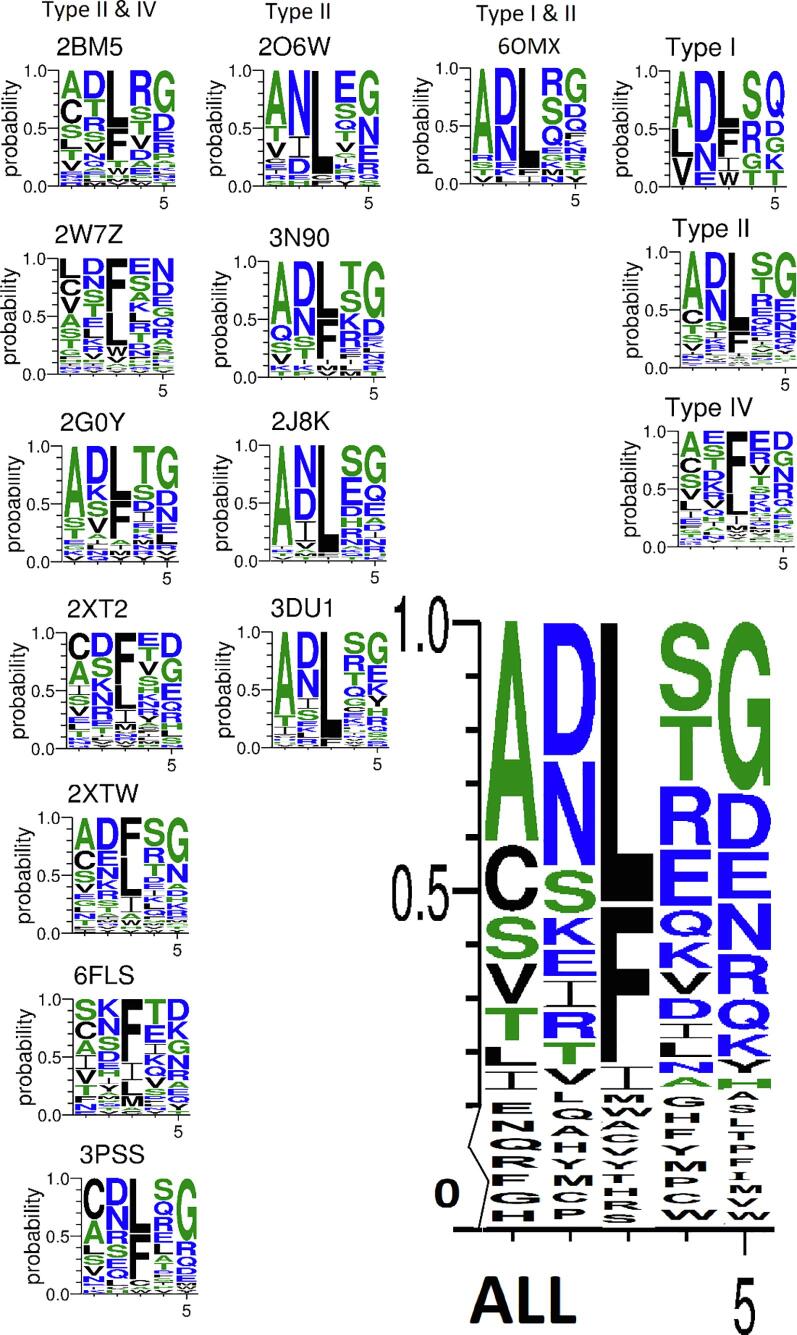
The sequence logo summary of all PRPs with known structures and alignments. The codes above each graph are the PDB code for each protein. The large sequence logo plot at the lower right was calculated using the all sequence alignment for all the other individual PRPs included in the figure.

Pentapeptide repeat domains have been reported to have the approximate consensus sequence (S/T/A/V)(D/N)(L/F)(S/T/R)(X) (Bateman et al., 1998, Vetting et al., 2006). However, prior to solving the crystal structure of Alr5209, we were able to predict the location of its pentapeptide repeat domains using this consensus sequence. Once the structure of Alr5209 was determined it was possible to map the pentapeptide repeat domains onto the Alr5209 amino acid sequence (Fig. 1). To facilitate reevaluation of the consensus sequences of pentapeptide repeat domains, a sequence Logo analysis was performed for all known PRP structures (Fig. 7). The sequence logo analysis in Fig. 7 is organized into representations for seven type I plus type IV β turn PRPs, four pure type II β turns PRPs and Alr5209, which is a mixture of type I and type II β turns. Based on our structure-based sequence analysis of all currently known PRP structures, we recommend that the consensus sequence of PRPs should be amended to (A/C/S/V/T/L/I)/(D/N/S/K/E/I/R)/(L/F)/(S/T/R/E/Q/K/V/D)/(G/D/E/N/R/Q/K). The complete list of the frequency of occurrence of every amino acid at every position in the pentapeptide repeat domain positions is compiled in Table S1. The general consensus from this analysis indicates that any uncharged or small hydrophobic amino acid can be accommodated in the i − 2 position, any charged or polar amino acid can be found in the i − 1 position, the i position is mostly occupied by L or F, followed by I, M, W, but can be occupied by any strongly hydrophobic residue, including A, C, V, the i + 1 positions can be occupied by any charged or polar amino acid, and the i + 2 positions can be occupied by any charged or polar amino acid. These rules are consistent with the topology of the PRPs in that the side chains of the i − 1 and i + 1 amino acids always point away from the axis of the right-handed β-helix, which in a water-soluble PRP would position the hydrophilic and charged side chains towards the solvent environment. Likewise, the side chain of the i position amino acid strongly prefers L or F to establish the hydrophobic core of the protein, but can also accommodate the side chain of any other hydrophobic amino acid. No charged amino acids have ever been observed in the i − 2 position, but uncharged, polar, hydrophilic amino acids have been observed in the i − 2 position.

### 3.7 Structural consequences of type I beta turns in PRPs

**Fig. 8:**
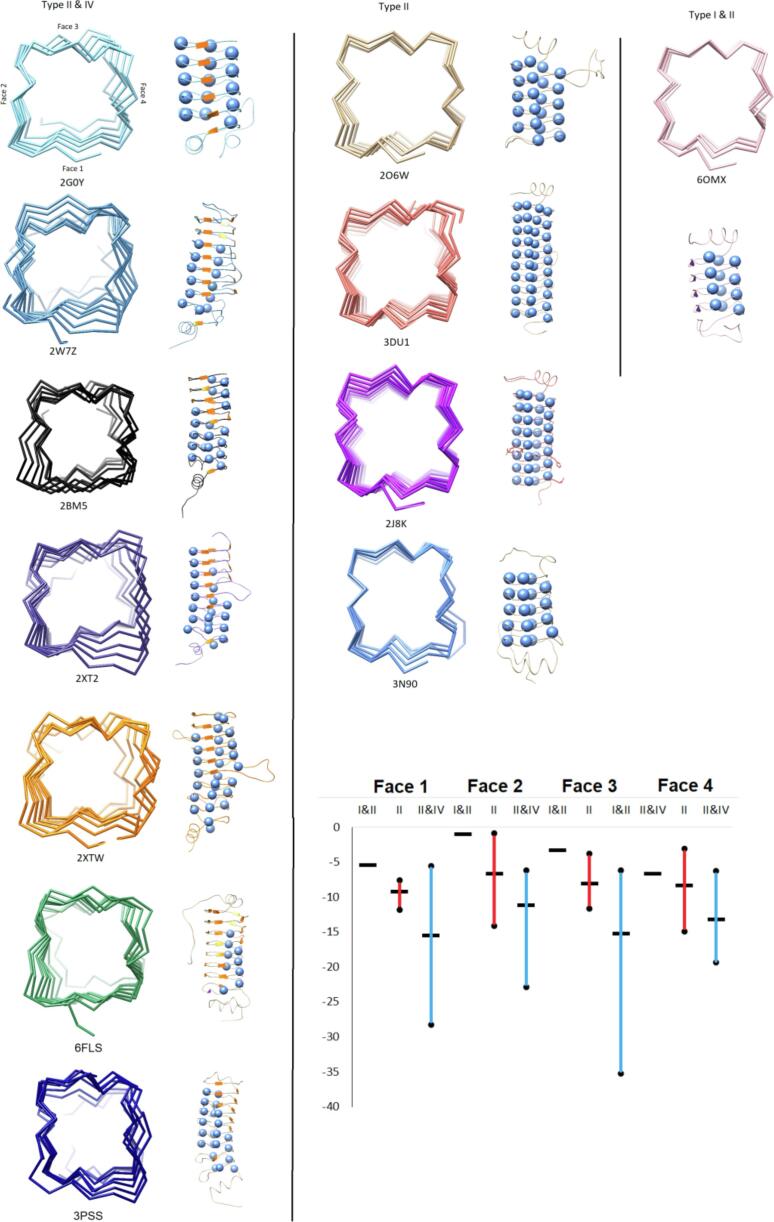
Backbone traces for all PRPs with known structures and sequence alignments. The PDB code is indicated below each structure. The first column shows all PRPs containing mixtures of type II and IV β turns with the turn distribution of turns indicated at the right where spheres indicate type II turns and orange sheets indicate type IV turns. The second column shows all PRPs made up exclusively of type II β turns. The last column shows Alr5209 which is the first example of a PRP that contains a mixture of type I and II β turns with the turn distribution indicated at the right where spheres indicate type II turns and purple sheets indicate type I turns. All structures are depicted with the N terminus facing the reader and the faces oriented the same as with 2G0Y with face 1 at the bottom, face 2 at the left, face3 at the top, and face 4 at the right. The inserted graph shows the average (bar) and range of twist angles of the three classes of PRPs based on their composition of β turns: type I plus type II, pure type II, or type II plus type IV as listed in Table 4. (For interpretation of the references to color in this figure legend, the reader is referred to the web version of this article.)

**Fig. 9:**
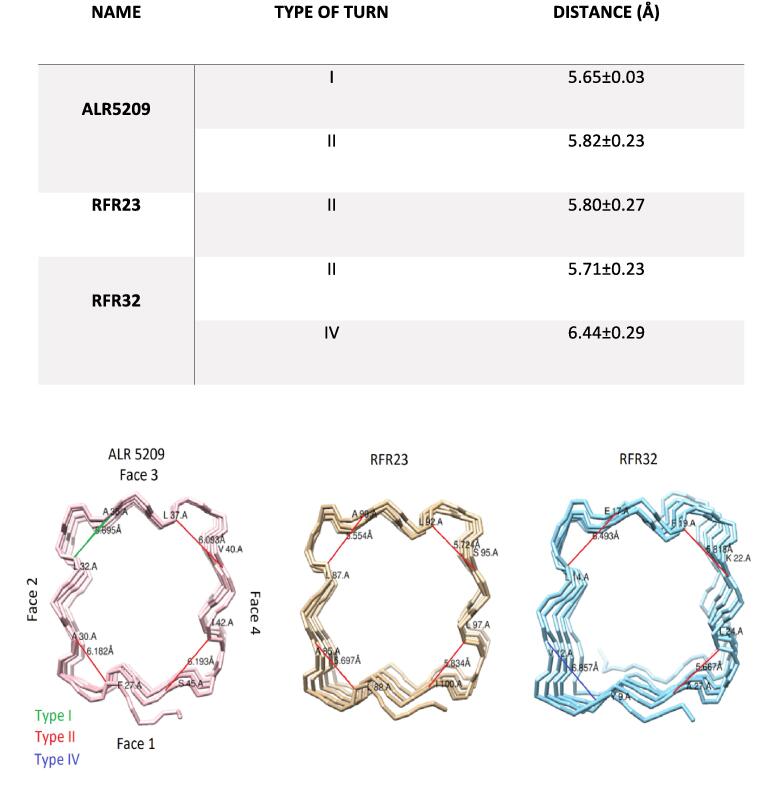
Graphs showing cross-turn distances for different types of turns and the summary of distance between carbon in i (i) and i − 2 (i + 1) position based on different types of turn. A) average distances across type I, type II and type IV β turns measured in three representative PRPs. B) Left) Distances measured across the β turns in Alr5209 (PDB ID 6OMX). Middle) Distances measured across the β turns in Rfr23 (PDB ID 2O6W). Right) Distances measured across the β turns in Rfr32 (PDB ID 2G0Y).

Based on analysis of all existing PRP structures in Protein Data Bank (Vetting et al., 2007, Buchko et al., 2007, Vetting et al., 2011a, Hegde et al., 2011, Xiong et al., 2011, Vetting et al., 2011b), Alr5209 is the first PRP that contains type I β turns in its Rfr fold. All other PRPs structures reported to date contain Rfr folds composed exclusively of type II β turns (206 W (Buchko et al., 2007), 3DU1 (Ni et al., 2009), 2J8K (Vetting et al., 2007), and 3N90 (Ni et al., 2011)) or mixture of type II and IV β turns (2G0Y (Buchko et al., 2006), 2W7Z (Hegde et al., 2011), 2BM5 (Hegde et al., 2005), 2XT2 (Vetting et al., 2011a), 2XTW (Vetting et al., 2011b), 6FLS (Notari et al., 2018), and 3PSS (Xiong et al., 2011)) (Fig. 7). In order to determine if there was any visible consequence of including type I β turns in the Rfr fold, we compared all existing PRP structures looking along the right-handed β-helical structure (Fig. 8). One pattern that is apparent is that PRPs composed of combinations of type II and IV β turns experience a significant negative twist in the relative position of the quadrilateral coils along the N-terminal to C-terminal direction (Fig. 8). PRPs comprised exclusively of type II β turns appear to also contain twist, but the magnitude of the negative twist is significantly smaller compared to PRPs containing both type II and type IV β turns (Fig. 8). Finally, Alr5209, composed of type I and type II β turns exhibits the least helical twist among known PRPs (Fig. 8). The twist angles for all PRPs are summarized in Table 4. Based on this analysis, increased magnitude of helical twist appears to be correlated with the presence of loops inserted into the pentapeptide repeat domain sequence, this being especially obvious among the combined type II and type IV β turn PRPs. However, when the twist magnitude was averaged on a per coil basis, the type II plus type IV β turn PRPs still had a significantly larger twist per coil magnitude (Fig. 9A), suggesting that a fundamental difference in the turn structure was responsible for introducing twist in the Rfr fold. To better understand the origin of increased negative twist in PRPs containing type IV β turns, the distances across each type of β turn were measured (Fig. 9B). These measurements indicated that the distance across type I β turns was the shortest at ~5.6 Å, compared to ~5.7–5.8 Å for type II β turns, however, the distance across type IV β turns was substantially longer at ~6.4 Å. Consequently, a negative helical twist is required to accommodate the extended β turn distance in comparison to the type I and type II β turn distances. Another consequence of the extended type IV β turns is a general increase in the area spanned by the individual quadrilateral coils (Table 5). This is evident both in the distance between the opposite faces of the quadrilateral β-helix, which increased by as much as 1 Å in going from type I plus type II β turn PRPs to type II plus type IV β turn PRPs (Table 5), and in the diagonal distances across the individual coils, which increased by about 1 Å in each direction (Table 5). Consequently, in this first example of a PRP comprised of both type I plus II β turns, the PRP solenoid is smaller and more compact with less negative helical twist compared to PRP structures made up exclusively of type II β turns and significantly smaller and more compact compared to PRP structures containing both type II and IV β turns, which, in general, have the largest Rfr folds.

**Table 4:** Summary of twist angles between coils for all PRPs with known structures.3.

β-Turn	PDB Code	Face 1(°)	Face 2(°)	Face 3(°)	Face 4(°)
Type I & II	6OMX	−5.5 ± 1.8^4^	−1.3 ± 1.6	−3.3 ± 2.1	−6.7 ± 2.9

Type II	3N90	−7.6 ± 2.7	−0.9 ± 0.5	−11.7 ± 5.5	−3.1 ± 3.7
	2J8K	−8.8 ± 3.5^4^	−6.8 ± 3.3	−8.9 ± 3.6	−8.3 ± 3.5
	2O6W	−8.7 ± 2.1	−4.7 ± 3.1	−3.8 ± 1.3	−7.0 ± 2.8
	3DU1	−11.8 ± 6.2^4^	−14.1 ± 6.4	−7.8 ± 7.3	−15.0 ± 7.8

Type II & IV	2XTW	−16.4 ± 6.5	−12.4 ± 6.9	−35.3 ± 12.5	−13.0 ± 8.4
	2W7Z	−28.2 ± 12.9	−6.2 ± 5.8	−6.2 ± 5.8	−6.3 ± 2.2
	2XT2	−26.4 ± 10.0	−2.8 ± 6.1	−21.5 ± 11.9	−16.1 ± 5.2
	2G0Y	−8.8 ± 2.5	−10.1 ± 5.7	−9.3 ± 2.4	−10.9 ± 4.7
	2BM5	−12.2 ± 5.2	−15.3 ± 6.4	−11.5 ± 5.1	−16.8 ± 7.7
	6FLS	−5.6 ± 3.1	−22.8 ± 12.2	−13.1 ± 5.6	−19.3 ± 6.4
	3PSS	−10.8 ± 5.6^4^	−8.2 ± 5.0	−9.9 ± 6.0	−9.9 ± 3.5

3 Twist angles are defined between first coil and following coils. Negative values negative twists and positive values indicate positive twists.

4 Angles compared starting from the second coil rather than the first coil.

**Table 5:** Summary of distances between and across faces of all PRPs with known structures and sequence alignments.^5^

β turn	PDB Code	Face 1 (Å)	Face 2 (Å)	Face 3 (Å)	Face 4 (Å)	Face 1–3 (Å)	Face 2–4 (Å)	Face 1–2 (Å)	Face 1–4 (Å)
Type I & II	6OMX	10.88 ± 0.02	10.41 ± 0.14	10.82 ± 0.14	11.09 ± 0.15	15.16 ± 0.25	14.41 ± 0.25	10.47 ± 0.36	10.95 ± 0.13

	3N90	11.12 ± 0.18	11.02 ± 0.22	10.95 ± 0.18	11.68 ± 1.11	15.43 ± 0.34	14.89 ± 0.82	10.82 ± 0.21	11.00 ± 0.39
	2J8K	11.05 ± 0.12	10.99 ± 0.12	10.92 ± 0.13	10.95 ± 0.18	14.77 ± 0.51	14.99 ± 0.31	10.51 ± 0.25	11.05 ± 0.13
Type II	2O6W	11.28 ± 0.37	11.06 ± 0.12	11.24 ± 0.18	10.88 ± 0.27	14.93 ± 0.38	15.51 ± 0.43	10.52 ± 0.13	11.82 ± 0.51
	3DU1	11.10 ± 0.12	11.09 ± 0.11	11.07 ± 0.22	11.05 ± 0.25	15.29 ± 0.50	15.02 ± 0.48	10.52 ± 0.23	11.40 ± 0.21
	Average	11.13 ± 0.22	11.04 ± 0.15	11.04 ± 0.22	11.10 ± 0.56	15.10 ± 0.53	15.08 ± 0.55	10.57 ± 0.24	11.33 ± 0.43

Type II &IV	2XTW	12.08 ± 0.53	11.44 ± 0.55	10.57 ± 1.27	10.95 ± 0.77	15.22 ± 0.54	11.08 ± 1.40	11.08 ± 0.87	12.09 ± 1.46
	2W7Z	11.84 ± 0.96	11.01 ± 0.39	11.40 ± 0.14	11.77 ± 0.76	16.01 ± 2.38	15.86 ± 1.27	11.20 ± 0.98	13.39 ± 0.88
	2XT2	11.79 ± 0.74	11.18 ± 0.29	10.92 ± 1.59	11.46 ± 0.72	15.71 ± 1.22	16.30 ± 0.50	10.74 ± 0.62	12.60 ± 0.64
	2G0Y	12.07 ± 0.29	11.24 ± 0.26	11.05 ± 0.18	10.99 ± 0.17	15.71 ± 0.23	15.45 ± 0.32	11.04 ± 0.13	12.04 ± 0.49
	2BM5	11.63 ± 0.57	11.54 ± 0.50	11.26 ± 0.49	10.98 ± 0.34	16.59 ± 0.91	15.14 ± 0.66	11.54 ± 0.92	11.87 ± 0.57
	6FLS	12.05 ± 0.42	12.26 ± 0.86	11.83 ± 0.47	11.37 ± 0.19	17.00 ± 1.37	16.69 ± 0.51	12.68 ± 0.79	12.37 ± 0.22
	3PSS	11.35 ± 0.57	10.70 ± 1.38	10.95 ± 0.37	11.90 ± 0.60	16.01 ± 1.51	15.19 ± 0.62	11.35 ± 0.57	12.14 ± 0.71
	Average	11.82 ± 0.68	11.35 ± 0.85	11.14 ± 0.95	11.37 ± 0.69	16.05 ± 1.49	15.83 ± 1.49	11.40 ± 0.98	12.35 ± 0.95

5 The distances of face 1,2,3,4 were measured from the carbonyl carbon of the first amino acids to that of the last amino acid. The distances between faces 1 and 3 were measured from the carbonyl carbon in face 1 i position to that in face 3 i position. The distances of face 2–4 are measured from the carbonyl carbon in face 2 i position to that in face 4 i position. The distances of face 1–2 are measured from the carbonyl carbon in face 1 i position to that in face 2 i position. The distances of face 1–4 are measured from the carbonyl carbon in face 1 i position to that in face 4 i position.

## 4 Conclusion

Alr5209 from *Nostoc* sp. PCC 7120 represents the first PRP structure that includes type I β turns in its Rfr fold. A combined analysis of its sequence and structure allowed us to investigate how type I β turns, along with type II and type IV β turns can be accommodated into Rfr folds, to characterize the consequences that the occurrence of type I β turns has on the right-handed β-helical coil structure, and to significantly expand our understanding of the consensus sequence observed in pentapeptide repeat protein domains. The thermal titration measurements obtained from CD experiments added to our understanding of how the relative thermal stability PRPs depends on the number of coils comprising the Rfr fold. While an understanding of the biochemical function of Alr5209 remains unknown, genomic analysis indicated that it may play a role in oxidative phosphorylation, however confirmation of such a role will require further examination.

## Declaration of Competing Interest

The authors declare that they have no known competing financial interests or personal relationships that could have appeared to influence the work reported in this paper.
