# Supplementary material for: Type I beta turns make a new twist in pentapeptide repeat proteins: Crystal structure of Alr5209 from Nostoc sp. PCC 7120 determined at 1.7 angström resolution
Source: J Struct Biol X. 2019 Aug 14;3:100010. doi: 10.1016/j.yjsbx.2019.100010 (PMC7337050; doi:10.1016/j.yjsbx.2019.100010)
Supplement: Supplementary data 1 [file mmc1.docx]

Supplementary Materials fr:

Type I Beta Turns Make A New Twist in Pentapeptide Repeat Proteins: Crystal Structure of Alr5209 from Nostoc sp. PCC 7120 Determined at 1.7 Angstr$\ddot{o}$m Resolution

Ruojing Zhang, Shuisong Ni, Michael A. Kennedy*

Department of Chemistry and Biochemistry, Miami University, Oxford, OH 45056

Contents:

**Table 1. Summary of amino acids distributions in all PRPs with known structures.**

**Table 1. Summary of amino acids distributions in all PRPs with known structures.**

|  |  | A | C | D | E | F | G | H | I | K | L | M | N | P | Q | R | S | T | V | W | Y |
| --- | --- | --- | --- | --- | --- | --- | --- | --- | --- | --- | --- | --- | --- | --- | --- | --- | --- | --- | --- | --- | --- |
|  | I-2 | 28 | 0 | 0 | 0 | 0 | 0 | 0 | 1 | 0 | 0 | 0 | 0 | 0 | 0 | 1 | 0 | 0 | 1 | 0 | 0 |
|  | I-1 | 1 | 0 | 9 | 0 | 0 | 0 | 0 | 5 | 0 | 0 | 1 | 12 | 0 | 0 | 0 | 0 | 1 | 2 | 0 | 0 |
| 2J8k | I | 0 | 0 | 0 | 0 | 1 | 0 | 0 | 1 | 0 | 29 | 0 | 0 | 0 | 0 | 0 | 0 | 0 | 0 | 0 | 0 |
|  | I+1 | 1 | 0 | 3 | 7 | 0 | 0 | 3 | 0 | 1 | 0 | 0 | 2 | 0 | 1 | 3 | 9 | 1 | 0 | 0 | 0 |
|  | I+2 | 2 | 0 | 2 | 4 | 0 | 10 | 1 | 2 | 1 | 0 | 0 | 2 | 0 | 5 | 2 | 0 | 0 | 0 | 0 | 0 |
|  | | | | | | | | | | | | | | | | | | | | | |
|  | I-2 | 10 | 1 | 0 | 1 | 0 | 0 | 0 | 1 | 0 | 0 | 0 | 0 | 0 | 0 | 1 | 1 | 3 | 3 | 0 | 0 |
|  | I-1 | 0 | 0 | 3 | 1 | 0 | 0 | 1 | 4 | 0 | 0 | 0 | 12 | 0 | 0 | 0 | 0 | 0 | 0 | 0 | 0 |
| 2O6W | I | 0 | 1 | 0 | 0 | 1 | 0 | 0 | 0 | 0 | 19 | 0 | 0 | 0 | 0 | 0 | 0 | 0 | 0 | 0 | 0 |
|  | I+1 | 0 | 0 | 0 | 6 | 0 | 1 | 0 | 1 | 1 | 0 | 0 | 0 | 1 | 2 | 1 | 3 | 2 | 2 | 0 | 1 |
|  | I+2 | 0 | 0 | 0 | 3 | 0 | 8 | 0 | 0 | 0 | 0 | 0 | 5 | 0 | 1 | 2 | 1 | 0 | 0 | 0 | 0 |
|  | | | | | | | | | | | | | | | | | | | | | |
|  | I-2 | 7 | 8 | 0 | 2 | 0 | 0 | 0 | 3 | 1 | 2 | 0 | 1 | 0 | 1 | 0 | 3 | 2 | 3 | 0 | 0 |
|  | I-1 | 0 | 0 | 7 | 2 | 0 | 0 | 0 | 1 | 4 | 1 | 0 | 4 | 0 | 0 | 4 | 6 | 2 | 1 | 0 | 1 |
| 2XT2 | I | 0 | 1 | 0 | 0 | 15 | 0 | 0 | 4 | 0 | 7 | 2 | 1 | 0 | 0 | 1 | 0 | 0 | 0 | 1 | 1 |
|  | I+1 | 1 | 1 | 0 | 4 | 1 | 0 | 2 | 1 | 2 | 1 | 1 | 2 | 1 | 0 | 2 | 3 | 4 | 4 | 0 | 2 |
|  | I+2 | 0 | 0 | 7 | 5 | 0 | 7 | 2 | 0 | 0 | 2 | 0 | 1 | 0 | 3 | 3 | 2 | 0 | 0 | 0 | 0 |
|  | | | | | | | | | | | | | | | | | | | | | |
|  | I-2 | 13 | 0 | 0 | 1 | 0 | 1 | 0 | 0 | 1 | 0 | 0 | 0 | 0 | 0 | 0 | 2 | 2 | 1 | 0 | 0 |
|  | I-1 | 1 | 0 | 7 | 0 | 0 | 0 | 0 | 1 | 3 | 1 | 0 | 1 | 0 | 1 | 0 | 3 | 0 | 3 | 0 | 0 |
| 2G0Y | I | 1 | 0 | 0 | 0 | 7 | 0 | 0 | 1 | 0 | 10 | 1 | 0 | 0 | 0 | 0 | 0 | 0 | 0 | 0 | 1 |
|  | I+1 | 0 | 0 | 2 | 1 | 0 | 0 | 1 | 2 | 1 | 0 | 1 | 1 | 0 | 0 | 1 | 3 | 7 | 0 | 0 | 1 |
|  | I+2 | 0 | 0 | 3 | 2 | 0 | 8 | 0 | 0 | 0 | 2 | 0 | 3 | 0 | 0 | 1 | 0 | 0 | 1 | 0 | 1 |
|  | | | | | | | | | | | | | | | | | | | | | |
|  | I-2 | 11 | 0 | 0 | 0 | 0 | 0 | 0 | 0 | 1 | 0 | 0 | 0 | 0 | 3 | 0 | 2 | 1 | 2 | 0 | 0 |
|  | I-1 | 0 | 0 | 8 | 0 | 0 | 0 | 0 | 1 | 1 | 0 | 0 | 5 | 1 | 0 | 0 | 2 | 2 | 0 | 0 | 0 |
| 3N90 | I | 0 | 0 | 0 | 0 | 7 | 0 | 0 | 1 | 0 | 10 | 1 | 0 | 0 | 0 | 0 | 0 | 0 | 1 | 0 | 0 |
|  | I+1 | 0 | 0 | 0 | 1 | 1 | 0 | 0 | 1 | 3 | 1 | 1 | 0 | 0 | 0 | 3 | 4 | 5 | 0 | 0 | 0 |
|  | I+2 | 0 | 0 | 3 | 1 | 0 | 10 | 0 | 0 | 1 | 1 | 0 | 1 | 0 | 1 | 1 | 0 | 1 | 0 | 0 | 0 |
|  | | | | | | | | | | | | | | | | | | | | | |
|  | I-2 | 9 | 7 | 0 | 1 | 0 | 0 | 0 | 0 | 0 | 3 | 0 | 1 | 0 | 1 | 1 | 4 | 3 | 2 | 0 | 0 |
|  | I-1 | 1 | 0 | 8 | 1 | 0 | 0 | 1 | 0 | 0 | 0 | 1 | 2 | 0 | 2 | 5 | 3 | 5 | 3 | 0 | 0 |
| 2BM5 | I | 0 | 0 | 0 | 0 | 9 | 0 | 1 | 0 | 0 | 16 | 1 | 0 | 0 | 0 | 0 | 0 | 2 | 2 | 1 | 0 |
|  | I+1 | 2 | 0 | 4 | 2 | 0 | 1 | 0 | 0 | 0 | 1 | 0 | 0 | 0 | 0 | 9 | 4 | 4 | 4 | 1 | 0 |
|  | I+2 | 1 | 0 | 5 | 3 | 0 | 12 | 1 | 0 | 1 | 1 | 0 | 1 | 2 | 1 | 3 | 0 | 0 | 1 | 0 | 0 |
|  | | | | | | | | | | | | | | | | | | | | | |
|  | I-2 | 29 | 1 | 0 | 0 | 0 | 0 | 0 | 3 | 0 | 0 | 0 | 0 | 0 | 0 | 1 | 1 | 4 | 1 | 0 | 0 |
|  | I-1 | 0 | 1 | 13 | 2 | 0 | 0 | 0 | 4 | 2 | 2 | 0 | 10 | 0 | 0 | 2 | 4 | 0 | 0 | 0 | 0 |
| 3DU1 | I | 0 | 0 | 0 | 0 | 3 | 0 | 0 | 0 | 0 | 37 | 0 | 0 | 0 | 0 | 0 | 0 | 0 | 0 | 0 | 0 |
|  | I+1 | 0 | 2 | 1 | 2 | 1 | 3 | 1 | 1 | 2 | 0 | 0 | 1 | 0 | 4 | 6 | 8 | 5 | 1 | 1 | 1 |
|  | I+2 | 1 | 0 | 1 | 6 | 0 | 11 | 3 | 0 | 4 | 0 | 0 | 1 | 0 | 2 | 3 | 2 | 0 | 0 | 0 | 4 |
|  | | | | | | | | | | | | | | | | | | | | | |
|  | I-2 | 11 | 7 | 0 | 2 | 1 | 1 | 0 | 1 | 0 | 2 | 0 | 2 | 0 | 0 | 0 | 4 | 2 | 3 | 0 | 0 |
|  | I-1 | 1 | 0 | 10 | 5 | 0 | 0 | 0 | 1 | 3 | 0 | 1 | 4 | 0 | 2 | 3 | 2 | 2 | 1 | 0 | 1 |
| 2XTW | I | 2 | 0 | 0 | 0 | 14 | 0 | 0 | 5 | 0 | 12 | 0 | 0 | 0 | 0 | 0 | 0 | 0 | 1 | 2 | 0 |
|  | I+1 | 0 | 0 | 2 | 2 | 1 | 1 | 0 | 2 | 2 | 2 | 0 | 0 | 0 | 2 | 5 | 9 | 4 | 1 | 1 | 0 |
|  | I+2 | 1 | 0 | 2 | 1 | 0 | 14 | 2 | 0 | 2 | 0 | 1 | 6 | 0 | 0 | 2 | 1 | 1 | 0 | 0 | 1 |
|  | | | | | | | | | | | | | | | | | | | | | |
|  | I-2 | 4 | 5 | 0 | 0 | 1 | 2 | 1 | 1 | 0 | 6 | 0 | 1 | 0 | 1 | 0 | 3 | 3 | 5 | 0 | 0 |
|  | I-1 | 0 | 0 | 5 | 3 | 0 | 0 | 1 | 1 | 2 | 3 | 0 | 4 | 0 | 1 | 2 | 4 | 4 | 2 | 0 | 1 |
| 2W7Z | I | 1 | 1 | 0 | 0 | 16 | 0 | 0 | 0 | 0 | 10 | 0 | 0 | 0 | 0 | 0 | 0 | 0 | 2 | 3 | 1 |
|  | I+1 | 3 | 0 | 2 | 5 | 1 | 0 | 1 | 0 | 3 | 3 | 0 | 2 | 1 | 1 | 3 | 5 | 3 | 1 | 0 | 0 |
|  | I+2 | 2 | 0 | 4 | 3 | 1 | 3 | 1 | 0 | 1 | 1 | 0 | 7 | 1 | 3 | 3 | 2 | 0 | 0 | 0 | 1 |
|  | | | | | | | | | | | | | | | | | | | | | |
|  | I-2 | 12 | 0 | 0 | 0 | 0 | 0 | 0 | 0 | 0 | 0 | 0 | 0 | 0 | 0 | 1 | 1 | 1 | 1 | 0 | 0 |
|  | I-1 | 0 | 0 | 8 | 1 | 0 | 0 | 0 | 0 | 1 | 1 | 0 | 5 | 0 | 0 | 0 | 0 | 0 | 0 | 0 | 0 |
| 6OMX | I | 0 | 0 | 0 | 0 | 1 | 0 | 0 | 1 | 0 | 14 | 0 | 0 | 0 | 0 | 0 | 0 | 0 | 0 | 0 | 0 |
|  | I+1 | 0 | 0 | 0 | 1 | 0 | 1 | 0 | 0 | 1 | 0 | 1 | 1 | 0 | 3 | 4 | 4 | 0 | 0 | 0 | 0 |
|  | I+2 | 0 | 0 | 2 | 0 | 1 | 5 | 0 | 0 | 1 | 0 | 0 | 1 | 0 | 2 | 1 | 1 | 1 | 0 | 0 | 1 |
|  | | | | | | | | | | | | | | | | | | | | | |
|  | I-2 | 4 | 5 | 0 | 1 | 2 | 0 | 0 | 4 | 0 | 0 | 0 | 2 | 0 | 0 | 0 | 6 | 3 | 4 | 0 | 0 |
|  | I-1 | 1 | 0 | 3 | 2 | 0 | 0 | 2 | 2 | 5 | 0 | 1 | 5 | 1 | 1 | 0 | 4 | 1 | 1 | 0 | 2 |
| 6FLS | I | 1 | 1 | 0 | 0 | 17 | 0 | 0 | 5 | 0 | 4 | 3 | 0 | 0 | 0 | 0 | 0 | 0 | 0 | 0 | 0 |
|  | I+1 | 0 | 0 | 1 | 5 | 1 | 0 | 0 | 3 | 3 | 1 | 0 | 1 | 0 | 3 | 0 | 2 | 7 | 3 | 0 | 1 |
|  | I+2 | 2 | 0 | 6 | 2 | 0 | 4 | 0 | 0 | 5 | 0 | 0 | 4 | 0 | 2 | 3 | 0 | 1 | 0 | 0 | 2 |
|  | | | | | | | | | | | | | | | | | | | | | |
|  | I-2 | 7 | 12 | 0 | 1 | 0 | 0 | 0 | 1 | 1 | 3 | 0 | 2 | 0 | 0 | 0 | 3 | 0 | 3 | 0 | 0 |
|  | I-1 | 0 | 1 | 8 | 3 | 0 | 0 | 1 | 0 | 0 | 2 | 0 | 7 | 0 | 3 | 4 | 4 | 0 | 0 | 0 | 0 |
| 3PSS | I | 1 | 2 | 0 | 0 | 13 | 0 | 0 | 0 | 0 | 16 | 0 | 0 | 0 | 0 | 0 | 0 | 0 | 0 | 1 | 0 |
|  | I+1 | 2 | 0 | 1 | 3 | 1 | 1 | 0 | 1 | 0 | 3 | 0 | 0 | 1 | 4 | 4 | 6 | 2 | 1 | 0 | 0 |
|  | I+2 | 0 | 0 | 2 | 2 | 0 | 17 | 0 | 0 | 0 | 0 | 1 | 0 | 0 | 3 | 4 | 0 | 0 | 0 | 1 | 1 |
|  | | | | | | | | | | | | | | | | | | | | | |
|  | I-2 | 4 | 0 | 0 | 0 | 0 | 0 | 0 | 0 | 0 | 2 | 0 | 0 | 0 | 0 | 0 | 0 | 0 | 2 | 0 | 0 |
|  | I-1 | 0 | 0 | 5 | 1 | 0 | 0 | 0 | 0 | 0 | 0 | 0 | 2 | 0 | 0 | 0 | 0 | 0 | 0 | 0 | 0 |
| Type I | I | 0 | 0 | 0 | 0 | 2 | 0 | 0 | 1 | 0 | 4 | 0 | 0 | 0 | 0 | 0 | 0 | 0 | 0 | 1 | 0 |
|  | I+1 | 0 | 0 | 0 | 0 | 0 | 1 | 0 | 0 | 0 | 0 | 0 | 0 | 0 | 0 | 2 | 3 | 1 | 0 | 0 | 0 |
|  | I+2 | 0 | 0 | 1 | 0 | 0 | 1 | 0 | 0 | 1 | 0 | 0 | 0 | 0 | 3 | 0 | 0 | 1 | 0 | 0 | 0 |
|  | | | | | | | | | | | | | | | | | | | | | |
|  | I-2 | 115 | 30 | 0 | 3 | 0 | 0 | 0 | 8 | 2 | 4 | 0 | 7 | 0 | 3 | 5 | 17 | 20 | 16 | 0 | 0 |
|  | I-1 | 2 | 1 | 74 | 6 | 0 | 0 | 1 | 15 | 11 | 6 | 3 | 65 | 1 | 3 | 11 | 19 | 5 | 5 | 0 | 2 |
| Type II | I | 4 | 5 | 0 | 0 | 43 | 0 | 0 | 8 | 0 | 159 | 3 | 0 | 0 | 0 | 1 | 0 | 1 | 3 | 4 | 0 |
|  | I+1 | 7 | 2 | 10 | 20 | 5 | 3 | 6 | 8 | 13 | 9 | 1 | 5 | 3 | 16 | 27 | 50 | 34 | 7 | 2 | 3 |
|  | I+2 | 3 | 0 | 19 | 27 | 2 | 94 | 6 | 2 | 11 | 3 | 1 | 18 | 1 | 13 | 14 | 5 | 1 | 0 | 1 | 10 |
|  | | | | | | | | | | | | | | | | | | | | | |
|  | I-2 | 25 | 15 | 0 | 6 | 4 | 4 | 1 | 7 | 2 | 10 | 0 | 2 | 0 | 3 | 0 | 14 | 4 | 11 | 0 | 0 |
|  | I-1 | 4 | 1 | 10 | 13 | 0 | 0 | 5 | 5 | 10 | 4 | 1 | 4 | 1 | 7 | 8 | 12 | 12 | 8 | 0 | 3 |
| Type IV | I | 2 | 1 | 0 | 0 | 59 | 0 | 1 | 9 | 0 | 20 | 5 | 1 | 0 | 0 | 0 | 0 | 1 | 3 | 3 | 3 |
|  | I+1 | 2 | 1 | 6 | 19 | 2 | 4 | 2 | 4 | 6 | 3 | 3 | 5 | 1 | 4 | 11 | 7 | 9 | 10 | 1 | 3 |
|  | I+2 | 6 | 0 | 17 | 5 | 0 | 14 | 4 | 0 | 4 | 4 | 1 | 14 | 1 | 7 | 14 | 4 | 2 | 2 | 0 | 1 |
|  | | | | | | | | | | | | | | | | | | | | | |
|  | I-2 | 144 | 46 | 0 | 9 | 4 | 4 | 1 | 15 | 4 | 16 | 0 | 9 | 0 | 6 | 5 | 31 | 24 | 29 | 0 | 0 |
|  | I-1 | 6 | 2 | 89 | 20 | 0 | 0 | 6 | 20 | 21 | 10 | 4 | 71 | 2 | 10 | 20 | 31 | 17 | 13 | 0 | 5 |
| All | I | 6 | 6 | 0 | 0 | 104 | 0 | 1 | 18 | 0 | 184 | 8 | 1 | 0 | 0 | 1 | 0 | 2 | 6 | 8 | 3 |
|  | I+1 | 9 | 3 | 16 | 39 | 7 | 8 | 8 | 12 | 19 | 12 | 4 | 10 | 4 | 20 | 41 | 60 | 44 | 17 | 3 | 6 |
|  | I+2 | 9 | 0 | 37 | 32 | 2 | 109 | 10 | 2 | 16 | 7 | 2 | 32 | 3 | 23 | 28 | 9 | 4 | 2 | 1 | 11 |
